# Role of the Renin Angiotensin System in Blood Pressure Allostasis-induced by Severe Food Restriction in Female Fischer rats

**DOI:** 10.1038/s41598-018-28593-6

**Published:** 2018-07-09

**Authors:** Aline Maria Arlindo de Souza, Crystal A. West, Aline Rezende Ribeiro de Abreu, Amrita V. Pai, Laura Batista Tavares Mesquita, Hong Ji, Deoclécio Chianca, Rodrigo Cunha Alvim de Menezes, Kathryn Sandberg

**Affiliations:** 1 Department of Medicine, Georgetown University, Washington, DC 20057 USA; 2 Departamento de Ciências Biológicas, Instituto de Ciências Exatas e Biológicas, Universidade Federal de Ouro Preto, Ouro Preto, MG 35460-000 Brazil; 3 Department of Biochemistry, Molecular and Cellular Biology, Georgetown University, Washington, DC 20057 USA; 4 Department of Psychiatry, Indiana University School of Medicine, Indianapolis, IN 46202 USA

## Abstract

Severe food restriction (FR) is associated with blood pressure (BP) and cardiovascular dysfunction. The renin-angiotensin system (RAS) regulates BP and its dysregulation contributes to impaired cardiovascular function. Female Fischer rats were maintained on a control (CT) or severe FR (40% of CT) diet for 14 days. In response to severe FR, BP allostasis was achieved by up-regulating circulating Ang-[1–8] by 1.3-fold through increased angiotensin converting enzyme (ACE) activity and by increasing the expression of AT_1_Rs 1.7-fold in mesenteric vessels. Activation of the RAS countered the depressor effect of the severe plasma volume reduction (≥30%). The RAS, however, still underperformed as evidenced by reduced pressor responses to Ang-[1–8] even though AT_1_Rs were still responsive to the depressor effects of an AT_1_R antagonist. The aldosterone (ALDO) response was also inadequate as no changes in plasma ALDO were observed after the large fall in plasma volume. These findings have implications for individuals who have experienced a period(s) of severe FR (e.g., anorexia nervosa, dieters, natural disasters) and suggests increased activity of the RAS in order to achieve allostasis contributes to the cardiovascular dysfunction associated with inadequate food intake.

## Introduction

Severe food restriction (FR) causes abnormalities in the heart, vascular system and kidneys and includes hypotension, bradycardia, mitral valve prolapse and a prolonged QT interval^1,2^. Other medical complications include edema, electrolyte disturbances (e.g., hypokalemia and hypophosphatemia), nephrolithiasis and kidney failure^3^. Severe FR can be self-imposed as a result of eating disorders like anorexia nervosa (AN)^4^ or in individuals who intentionally reduce their percent body fat for their occupation (e.g., models, athletes, dancers) or other reasons (e.g., dieters). Non-voluntary FR occurs in situations when people have inadequate access to food such as in war, natural disasters, periods of famine or low socio-economic conditions^5,6^. Severe FR can also occur in illnesses like cancer and AIDS due to inadequate food intake as a result of nausea or low palatability^7,8^. In these cases, the malnutrition as a result of the severe FR can independently add to the health complications of the disease.

We have shown that female Fischer rats maintained on a severe FR diet for 14 days (40% of a normal diet) developed malnutrition and shared numerous biochemical and physiological parameters found in humans after severe FR^9^. FR rats exhibited increased sensitivity to cardiopulmonary reflexes, the Bezold-Jarisch reflex, and α1-adrenoreceptor responses. We and others have also shown this model of FR leads to the development of cardiovascular abnormalities including ventricular hypertrophy and hypotension^9,10^.

The renin-angiotensin system (RAS) plays a critical role in blood pressure (BP) homeostasis and fluid and electrolyte balance and is a central regulator of cardiovascular and renal function. The activity of the octapeptide hormone angiotensin II (Ang-[1–8]) is widely targeted clinically in the treatment of hypertension and numerous cardiovascular and kidney diseases. In addition to its peripheral and central effects, Ang-[1–8] can act as a paracrine, autocrine, and intracrine hormone^11,12^. Ang-[1–8] exerts its vasoconstrictor effects by binding to angiotensin type 1 receptors (AT_1_R) in resistance vessels. Ang-[1–8] also acts in the brain to influence responses that modulate BP including water intake and the sympathetic nervous system^13^. In addition, Ang-[1–8] directly increases sodium and water reabsorption in the kidney and stimulates ALDO release from the zona glomerulosa of the adrenal gland^14^.

Animal studies of low protein isocaloric diets^15^ and chronic undernutrition^16^ implicate Ang-[1–8] in the cardiovascular dysfunction associated with these conditions. Ang-[1–8] dysfunction is also implicated in the cardiovascular abnormalities associated with AN. Women with AN exhibited reduced BP responses to Ang-[1–8]^17^. To further understand the impact of FR on the regulation of the RAS in the female, we investigated the effect of a severe FR diet in the female Fischer rat on the major peptides, receptors and enzymes of the RAS as well as BP and HR responses to RAS peptides and an antagonist of the AT_1_R.

## Results

### Hemodynamic parameters and urine and plasma measurements

**Figure 1:**
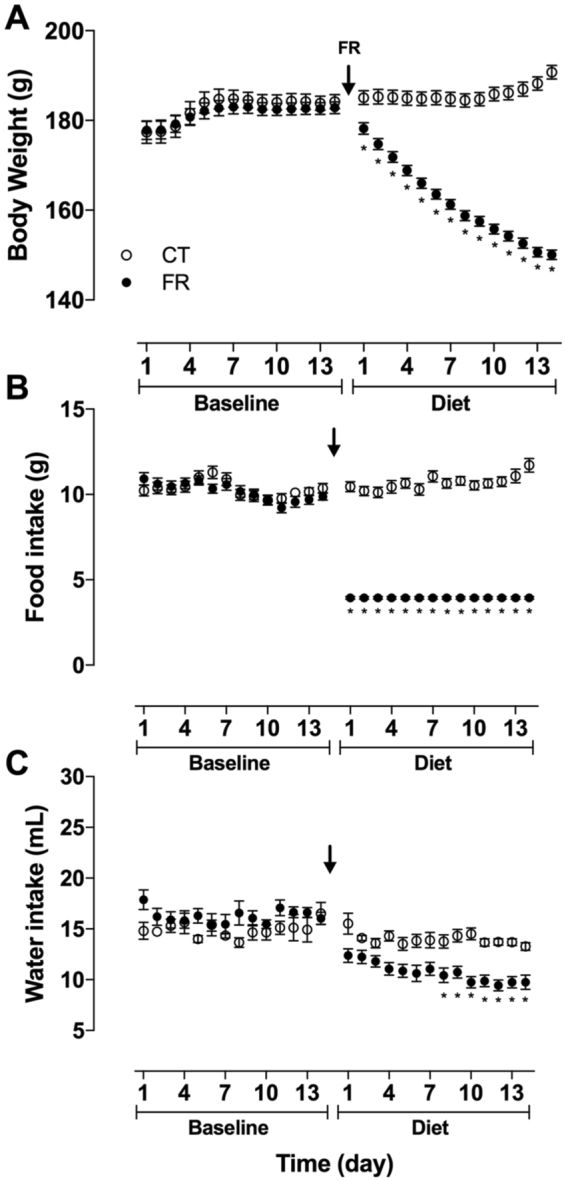
Effect of FR on BW and food and water intake. Shown is daily BW (CT, n = 34; FR, n = 41) (**A**), food (CT, n = 34; FR, n = 41) (**B**) and water (CT = 10; FR = 16) (**C**) intake in female rats before (baseline) and after maintenance on a CT (open circle) or FR (closed circle) diet. ^*^p < 0.05 vs. CT, by two-way ANOVA and Bonferroni post-hoc test (time; diet). Values are expressed as the mean ± SEM.

Before beginning the FR protocol, there was no difference in initial body weight (BW) between the CT (184 ± 2 g) and FR (183 ± 1 g) animal groups or in daily food intake. Over 2 weeks, the CT group slowly gained weight while BW in the FR group continued to fall; by day 14, BW in the FR rats was 80% of the CT group (Fig. 1A). Daily food intake in the FR group was 40% of the CT animals (Fig. 1B). Daily water intake remained similar throughout the study in the CT group whereas after 8 days on the FR diet, daily water intake was 30% less than CT animals (Fig. 1C).

Basal MAP and HR and plasma volume were lower in the FR group (Table 1) as we previously found^9,10^ and as observed in self-imposed cases of FR in humans^1,2^. The hematocrit was higher in the FR compared to the CR group, which is also similar to what is found in individuals suffering from low food intake^18^. FR reduced urine volume by greater than 30% but had no effect on plasma ALDO or plasma potassium though plasma sodium showed a trend towards being elevated (Table 1). FR also caused reductions in sodium and potassium intake and excretion (U_Na_V, U_K_V) although net sodium (Na+ intake – UnaV) and potassium balance (K^+^ intake - U_K_V) were maintained (Table 1). Under lengthier or more severe FR conditions, sodium and potassium balance can become impaired^3^, however, in our experimental conditions, we found FR for 14 days did not impair sodium or potassium balance. Thus, this protocol represents a moderate model of metabolic dysfunction.

**Table 1:** Effect of FR on hemodynamic and urine and plasma parameters.

	CT	FR	P value (<)
	Mean ± SEM, N	Mean ± SEM, N	
MAP (mmHg)	113 ± 1.9, n = 33	107 ± 1.6, n = 32	**0.03** ^**‡**^
HR (bpm)	380 ± 5.8, n = 33	357 ± 8.9, n = 32	**0.03** ^**‡**^
Plasma Aldosterone (pg/mL)	783 ± 123, n = 8	1020 ± 155, n = 7	0.2
Hematocrit (fraction)	0.45 ± 0.002, n = 7	0.48 ± 0.007, n = 6	**0.001** ^**‡**^
Plasma volume (mL)	9.0 ± 1.0, n = 8	6.1 ± 0.7, n = 8	**0.03** ^**‡**^
Urine volume (mL/24 h)	11.6 ± 0.7, n = 8	5.0 ± 0.3, n = 8	**0.001** ^**‡**^
Na^+^ intake (mmol/24 h)	1.893 ± 0.087, n = 8	0.673 ± 0.002, n = 8	**0.001** ^**‡**^
U_Na_ (mmol/L)	165.7 ± 4.7, n = 8	141.6 ± 11.7, n = 8	0.09
U_Na_V (mmol/24 h)	1.92 ± 0.10, n = 8	0.70 ± 0.045, n = 8	**0.001** ^**‡**^
Na^+^ intake - U_Na_V (mmol/24 h)	−0.05 ± 0.05, n = 8	−0.05 ± 0.05, n = 8	0.99
Plasma Na^+^ concentration (mmol/L)	138.7 ± 0.9, n = 8	142.3 ± 1.4, n = 8	0.056
K^+^ intake (mmol/24 h)	4.033 ± 0.18549, n = 8	1.430 ± 0.0051, n = 8	**0.001** ^**‡**^
U_K_ (mmol/24 h)	361 ± 1130, n = 8	321 ± 1232, n = 8	**0.04** ^**‡**^
U_K_V (mmol/24 h)	4.15 ± 0.164, n = 8	1.59 ± 0.051, n = 8	**0.001** ^**‡**^
K^+^ intake - U_K_V (mmol/24 h)	−0.19 ± 0.113, n = 8	−0.22 ± 0.051, n = 8	0.8
Plasma K^+^ concentration (mmol/L)	5.3 ± 0.26, n = 8	4.9 ± 0.13, n = 8	0.3
Urine protein (mg/mL)	10.7 ± 0.41, n = 8	11.8 ± 0.71, n = 7	0.2

Basal physiological values in female rats subjected to control (CT) or food restricted (FR) diet for 14 days. ^**‡**^P < 0.05 vs CT, by unpaired Student’s *t-*test. BW, body weight; MAP, mean arterial pressure; HR, heart rate; U_Na_V, urinary excretion of Na^+^; U_K_V, urinary excretion of K^+^.

### Ang-[1–10], Ang-[1–8] and Ang-[1–7] pressor and HR responses

**Figure 2:**
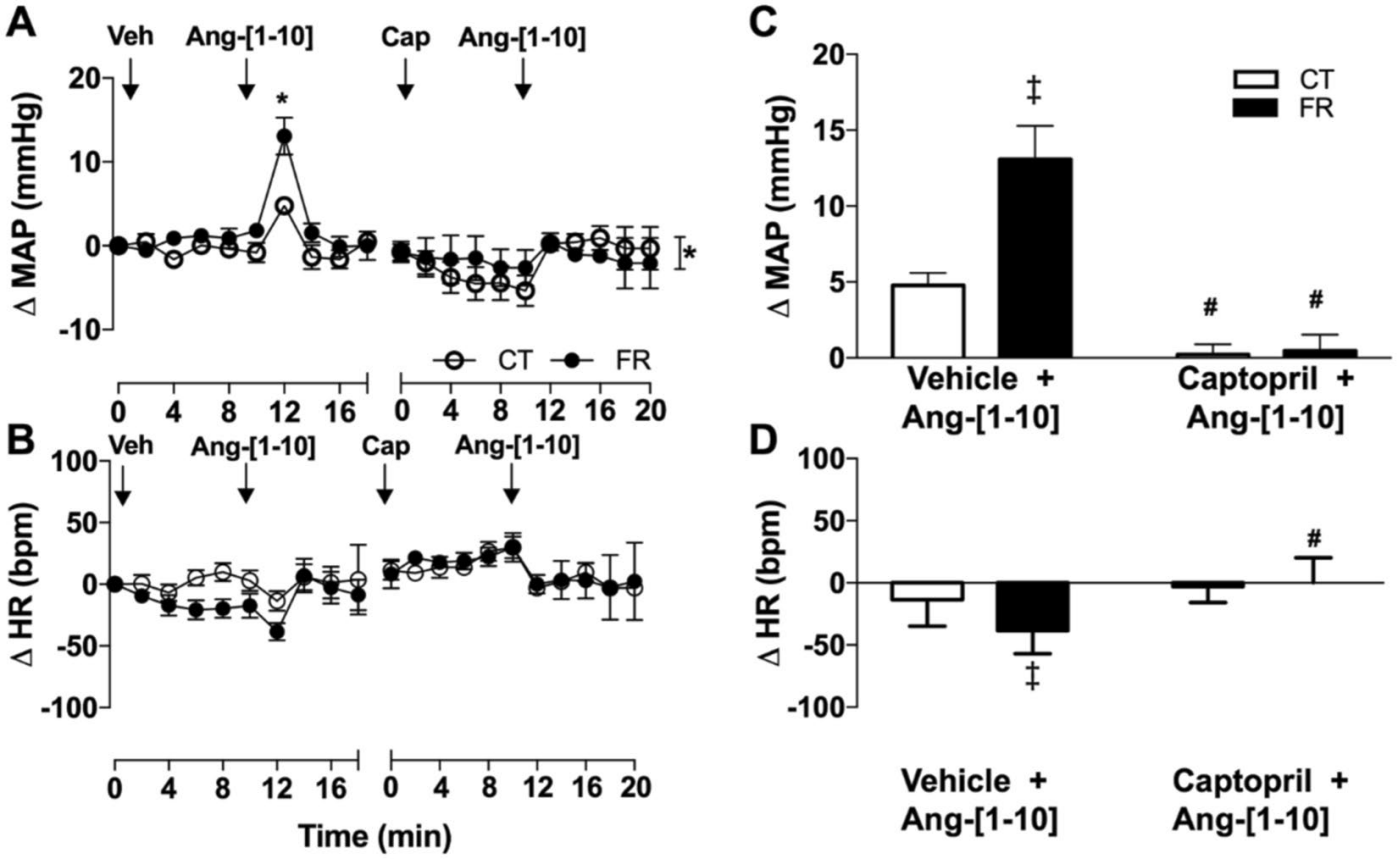
Effect of FR on MAP and HR responses to infusion of Ang-[1–10]. Shown are the changes (**A**,**B**) or maximum changes from baseline (**C**,**D**) in MAP (**A**,**C**) and HR (**B**,**D**) after vehicle (Veh), Ang-[1–10] or captopril (Cap) infusion in CT (open circle) and FR (closed circle) female rats (n = 7/group). Basal MAP (mm Hg): CT, 106 ± 1.3 vs. FR, 104 ± 3.0. Basal HR (bpm): CT, 368 ± 13 vs. FR, 355 ± 14. *P < 0.05 vs. CT by two-way ANOVA and Bonferroni post-hoc test (time; diet); ^#^P < 0.001 vs. vehicle, same animal group by unpaired Student’s *t*-test and ^‡^p < 0.002 vs. CT, same treatment, by two-way ANOVA and Bonferroni post-hoc test (drug; diet). Values are expressed as the mean ± SEM.

**Figure 3:**
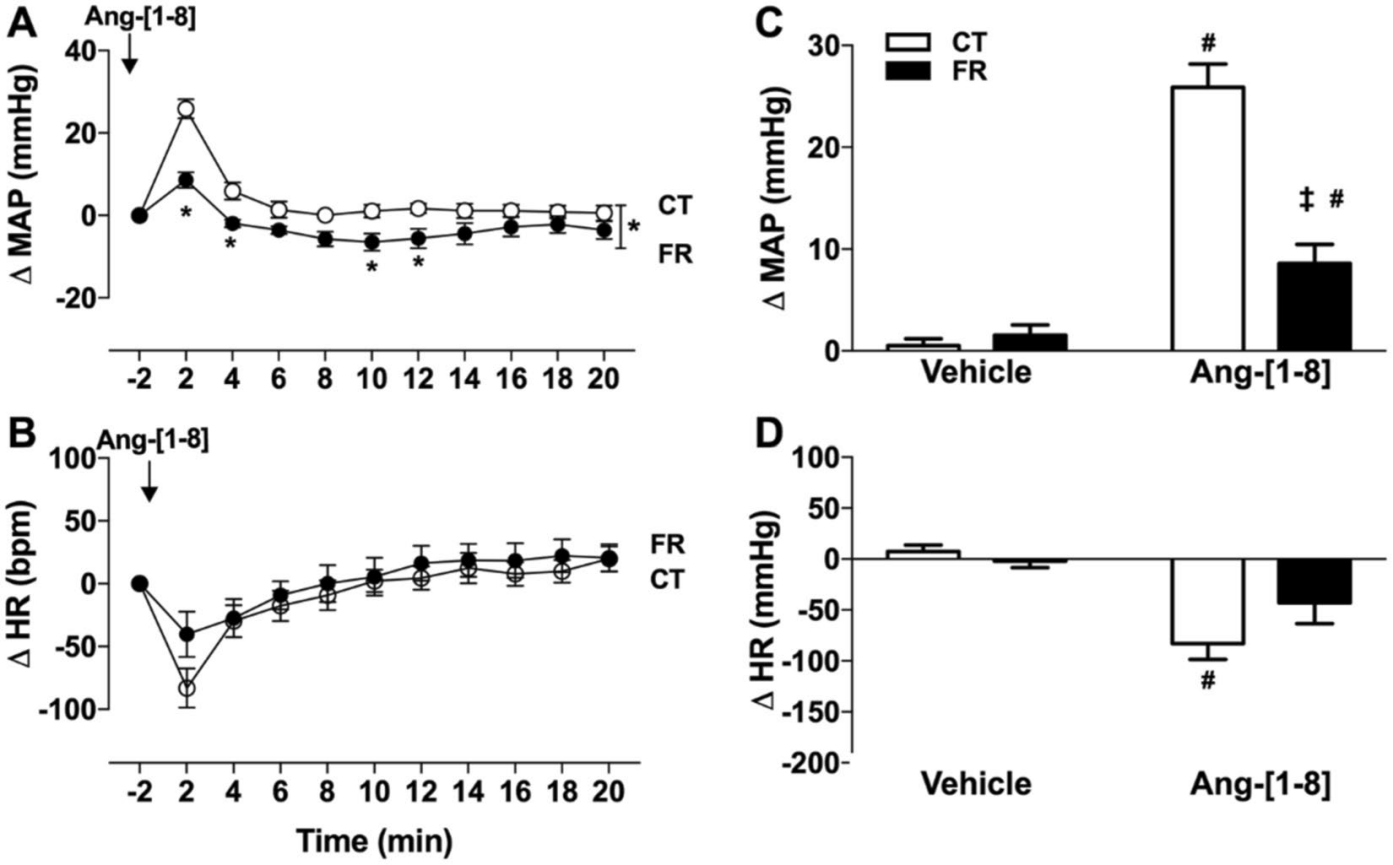
Effect of FR on MAP and HR responses to infusion of Ang-[1–8]. Shown are the changes (**A**,**B**) or maximum change from baseline (**C**,**D**) in MAP (**A**,**C**) and HR (**B**,**D**) after vehicle or Ang-[1–8] infusion in CT (open circle) and FR (closed circle) female rats (n = 8/group). Basal MAP (mm Hg): CT, 117 ± 5.0 vs. FR, 114 ± 5.0; Basal HR (bpm): CT, 386 ± 14 vs. *FR, 309 ± 18. *P < 0.001 vs. CT by two-way ANOVA and Bonferroni post-hoc test (time; diet); ^#^P < 0.01 vs. vehicle, same animal group by unpaired Student’s *t*-test and ^‡^p < 0.001 vs. CT, same treatment, by two-way ANOVA and Bonferroni post-hoc test (drug; diet). Values are expressed as the mean ± SEM.

**Figure 4:**
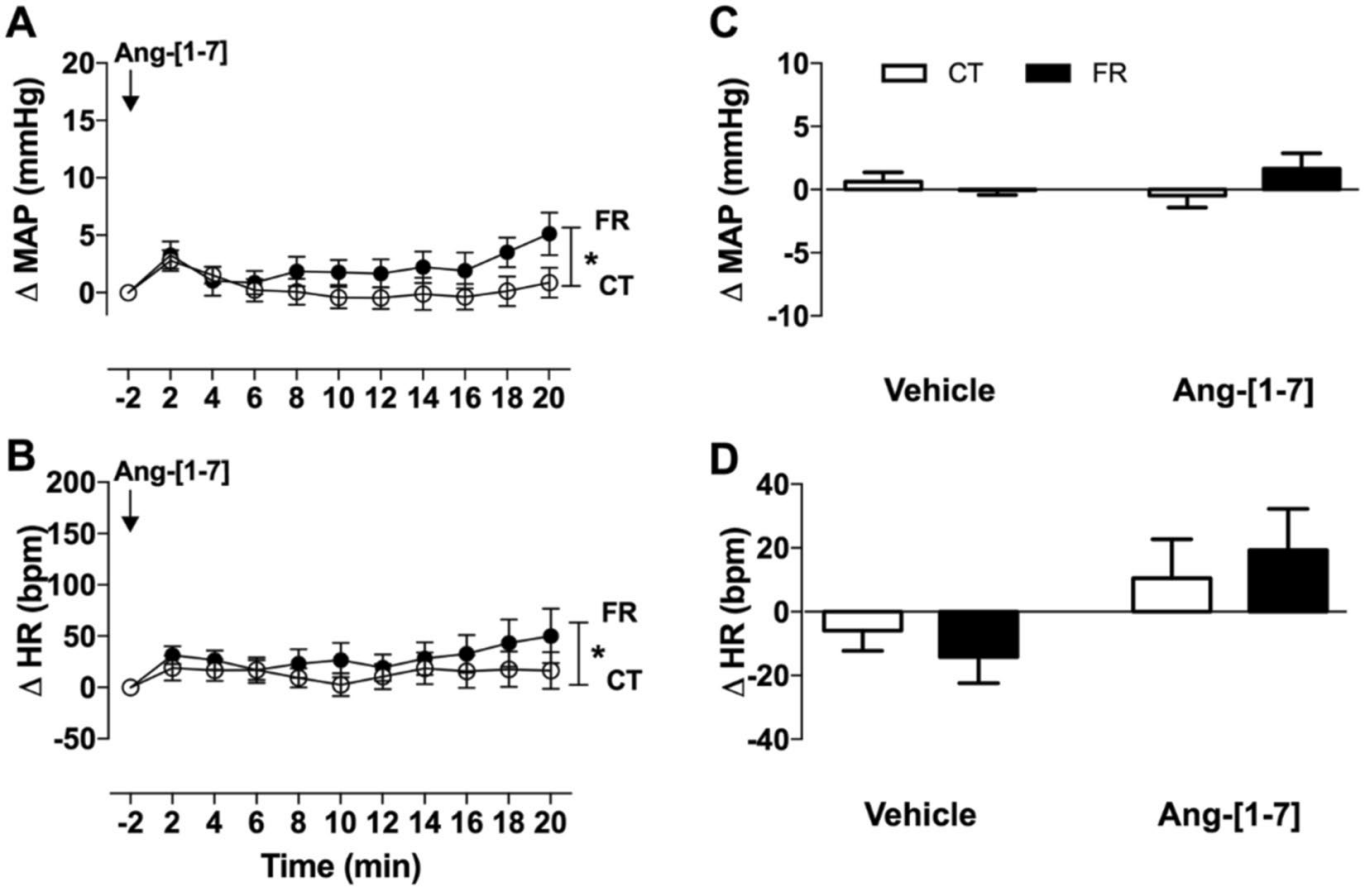
Effect of FR on MAP and HR responses to infusion of Ang-[1–7]. Shown are the changes (**A**,**B**) or maximum change from baseline (**C**,**D**) in MAP (**A**,**C**) and HR (**B**,**D**) after vehicle or Ang-[1–7] infusion in CT (open circle; n = 10) and FR (closed circle; n = 9) female rats. Basal MAP (mm Hg): CT, 106 ± 1 vs. FR, 104 ± 3. Basal HR (bpm): CT, 380 ± 7 vs. FR, 358 ± 12. *P < 0.05 vs. CT by two-way ANOVA and Bonferroni post-hoc test (time; diet). Values are expressed as the mean ± SEM.

To study the impact of FR on RAS hemodynamic regulatory mechanisms, we measured BP and HR responses to Ang-[1–10]. FR increased the pressor (Fig. 2A,C) and reduced the HR (Fig. 2B,D) responses to Ang-[1–10] infusion. Pretreatment with the angiotensin converting enzyme (ACE) inhibitor, captopril prevented the pressor and HR responses to Ang-[1–10] in both the FR and CT groups. FR attenuated the pressor (Fig. 3A,C) and HR (Fig. 3B,D) responses to Ang-[1–8] infusion. FR altered the time course of MAP (Fig. 4A,C) and HR (Fig. 4B,D) responses to Ang-[1–7] infusion without affecting peak MAP and HR responses.

### Plasma ACE, ACE2 and renin activity

**Figure 5:**
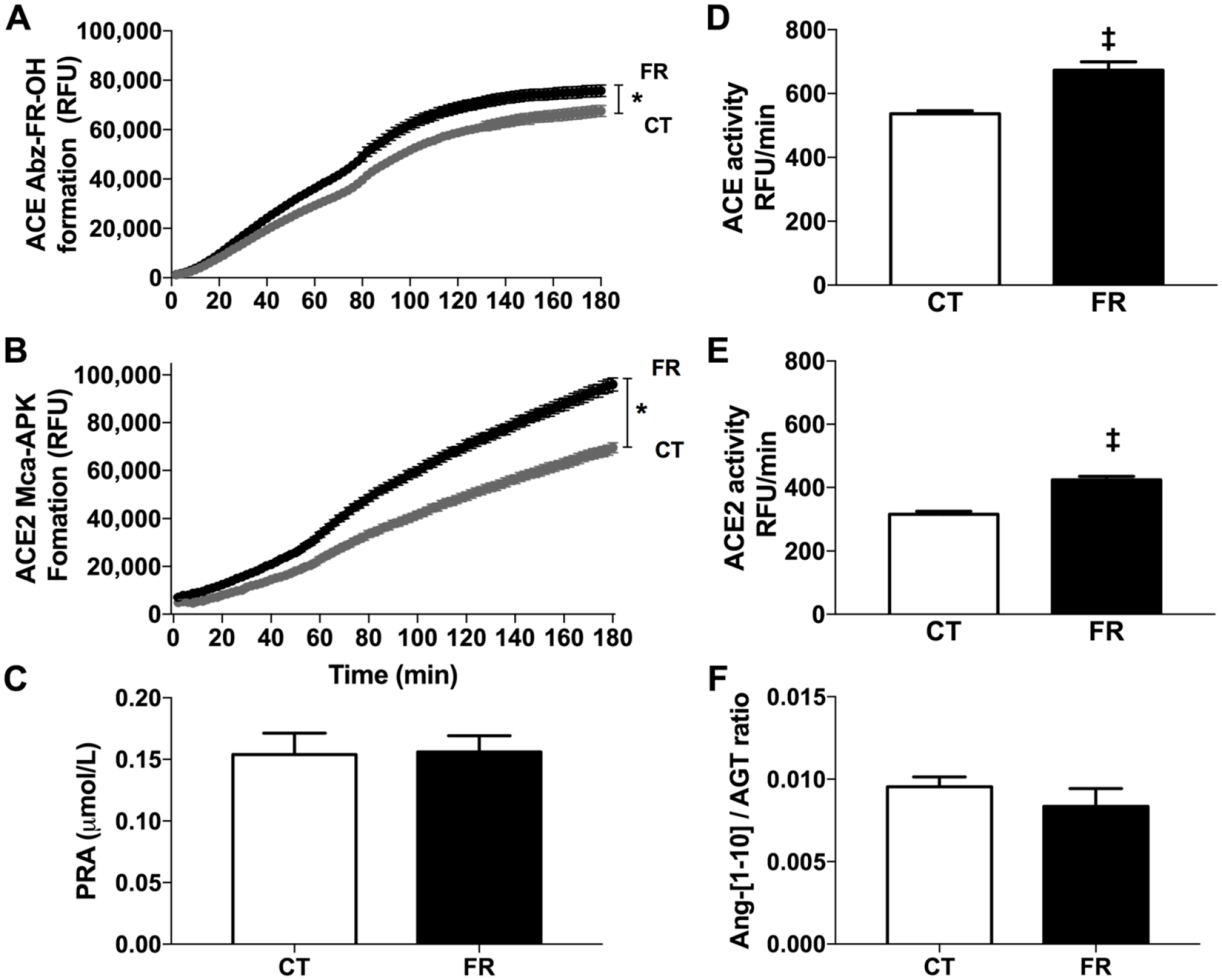
Effect of FR on plasma ACE, ACE2 and renin activity. Shown is the time course of product formation (**A**,**B**) and enzyme activity (**C**,**D**,**E**) for ACE (n = 8/group) (**A**,**D**), ACE2 (CT, n = 8; FR = 9) (**B**,**E**) and renin (CT, n = 8; FR, n = 10) **(C)** in the plasma and the ratio of Ang-[1–10] over AGT production (CT, n = 10; FR, n = 11) (**F**) in CT (grey circle; white bar) and FR (closed circle; black bar) female rats. *P < 0.001 vs. CT by two-way ANOVA and Bonferroni post-hoc test (time; diet); ^‡^P < 0.0001 vs. CT, same treatment, by unpaired Student’s *t*-test. Values are expressed as the mean ± SEM.

The major catabolic enzymes of the RAS include renin, ACE and ACE2. Renin acts upon angiotensinogen to produce the inactive decapeptide Ang-[1–10]. ACE then catabolizes Ang-[1–10] to produce the vasoconstrictor Ang-[1–8]. ACE2 removes one amino acid Ang-[1–8] to form the vasodilator Ang-[1–7]. To assess the effects of FR on key metabolic pathways of the RAS, renin activity and ACE and ACE2 were determined in the plasma. The rate of product formation produced by ACE (Fig. 4A,D) and ACE2 (Fig. 5B,E) was faster in the plasma of FR compared to CT animals. In contrast, FR had no effect on plasma renin activity (Fig. 5C) and the ratio of plasma Ang-[1–10]/AGT (Fig. 5F).

### Plasma AGT and Ang peptides

**Figure 6:**
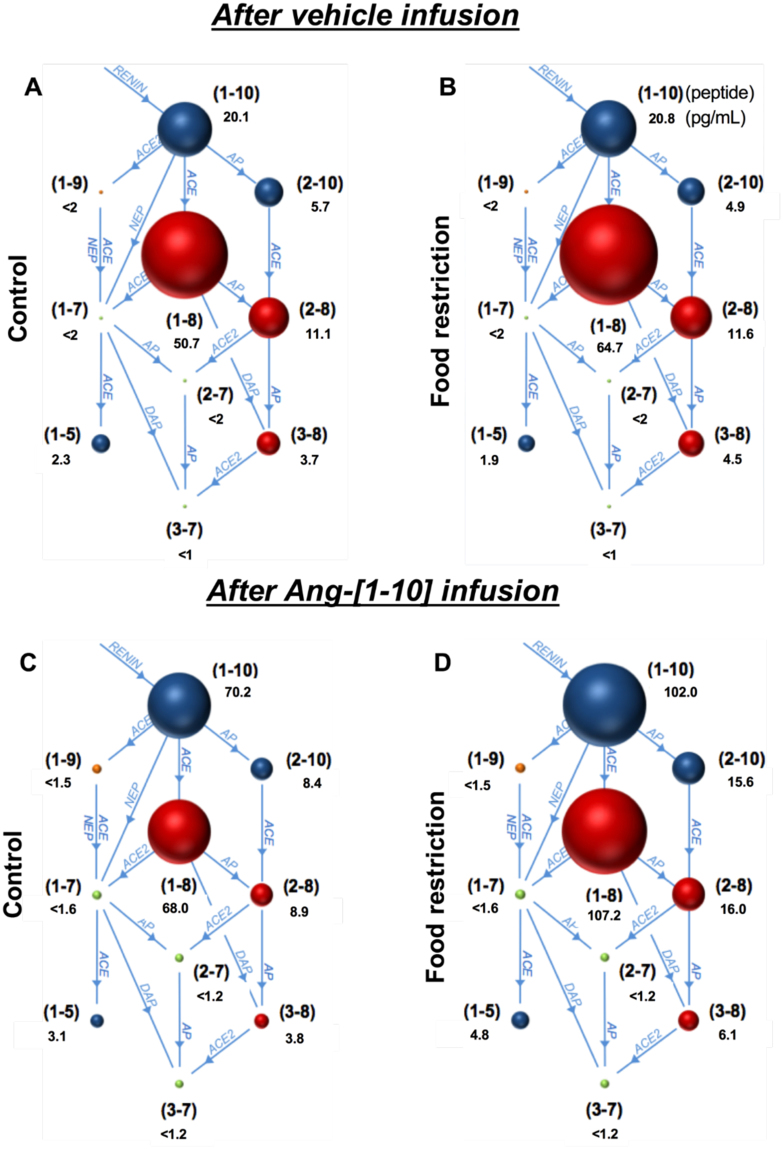
Depiction of the relative effect of FR on plasma angiotensin (Ang) peptides. Shown is the RAS fingerprint of Ang peptides in CT (n = 10) (**A**,**C**) and FR (n = 11) (**B**,**D**) female rats before (**A**,**B**) and after Ang-[1–10] infusion (**C**,**D**) (n = 8/group). The numbers in parentheses refer to the specific Ang peptide with the peptide concentration (pg/mL) listed below each peptide. For example, (1–10) represents Ang-[1–10]. Red and green circles reflect biologically active and inactive Ang peptides, respectively. The relative amounts of plasma peptides are depicted by the circle volumes, which are drawn to a relative scale.

To further assess the effects of FR on the RAS metabolic pathway, angiotensin peptides were measured by the RAS fingerprint® method. FR increased plasma levels of AGT and Ang-[1–8] (Table 2, Fig. 6). FR had no effect on basal plasma levels of Ang-[1–10], Ang-[2–10], Ang-[2–8], Ang-[3–8] and Ang-[1–5]. The other Ang peptides were below the limits of detection; however, after Ang-[1–10] infusion, AGT, Ang-[1–10], Ang-[1–8], Ang-[1–5], Ang-[2–10], Ang-[2–8], and Ang-[3–8] were increased to a greater extent in the FR compared to the CT group.

**Table 2:** Effect of food restriction on plasma angiotensin peptides before and after Ang-[1–10] infusion.

Peptide	Vehicle infusion			Ang-[1–10] infusion			Vehicle *vs* Ang-[1–10]	
	CT (pg/mL±SEM)	FR (pg/mL±SEM)	P value	CT (pg/mL±SEM)	FR (pg/mL±SEM)	P value	CT P value	FR P value
AGT (ng/mL)	2086 ± 72	2492 ± 24	**<0.001** ^‡^	1810 ± 65	2656 ± 70	**<0.001** ^‡^	**0.01** ^**#**^	**0.04** ^**#**^
Ang-[1–10]	20.1 ± 1.3	20.8 ± 2.7	0.99	70.2 ± 9.5	102 ± 8.7	**<0.005** ^**‡**^	**<0.001** ^**#**^	**<0.001** ^**#**^
Ang-[2–10]	5.9 ± 0.6	5.0 ± 0.5	0.96	8.4 ± 0.8	16 ± 2.1	**<0.001** ^**‡**^	**<0.02**	**<0.001** ^**#**^
Ang-[1–9]	<1.7 ± 0.04	<1.6 ± 0.05	nd	<1.5 ± 0.1	<1.4 ± 0.03	nd	nd	nd
Ang-[1–8]	50.7 ± 4.9	64.6 ± 3.5	**<0.05** ^**‡**^	67.9 ± 7.1	107 ± 16	**<0.05** ^**‡**^	0.055	**<0.01** ^**#**^
Ang-[1–7]	<2.2 ± 0.04	<2.1 ± 0.05	nd	<1.6 ± 0.03	<1.6 ± 0.04	nd	nd	nd
Ang-[2–8]	11.1 ± 1.2	11.5 ± 1.3	0.99	8.9 ± 1	16.0 ± 2	**<0.02** ^**‡**^	0.26	0.12
Ang-[3–8]	3.7 ± 0.5	4.5 ± 0.3	0.42	3.8 ± 0.3	6.1 ± 0.6	**<0.005** ^**‡**^	0.90	**<0.05** ^**#**^
Ang-[2–7]	<2.3 ± 0.2	<1.9 ± 0.2	nd	<1.2 ± 0.04	<1.1 ± 0.04	nd	nd	nd
Ang-[3–7]	<1.2 ± 0.02	<1.2 ± 0.02	nd	<1.2 ± 0.04	<1.1 ± 0.03	nd	nd	nd
Ang-[1–5]	2.3 ± 0.2	2.0 ± 0.2	0.88	3.1 ± 0.1	4.8 ± 0.6	**<0.01** ^**‡**^	<0.02^#^	**<0.001** ^**#**^

Blood angiotensinogen (n = 8/group) and angiotensin (Ang) peptide concentrations before (CT, n = 10; FR, n = 11/group) and after Ang-[1–10] infusion (n = 8/group) in female rats subjected to control (CT) or food restricted (FR) diet for 14 days. ^‡^P < 0.05 vs CT by two-way ANOVA and Bonferroni post-hoc test (drug; diet); ^#^P < 0.05 vs vehicle, same group^,^ by unpaired Student’s *t*-test. (**<**) in front of a number indicates below threshold of signal-to-noise; nd, not detectable.

### AT_1_R, AT_2_R, and MasR mRNA expression in mesenteric vessels

**Figure 7:**
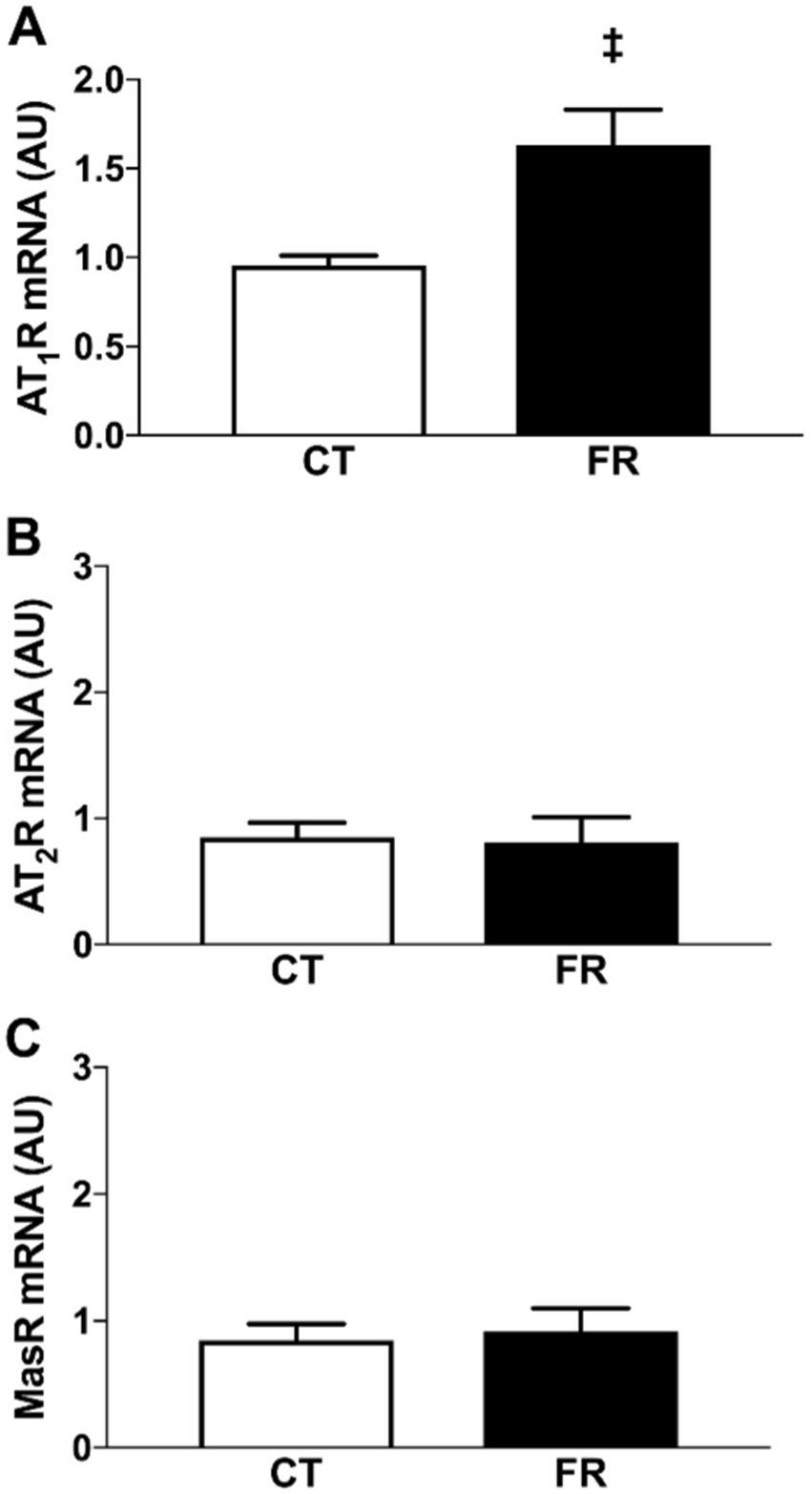
Effect of FR on AT_1_R, AT_2_R and MasR mRNA in mesenteric vessels. Shown is the basal mRNA expression levels of the AT_1_R (**A**), AT_2_R (**B**) and MasR (**C**) in mesenteric vessels from CT (white bar; n = 8) and FR (black bar; n = 9) female rats. ^‡^P < 0.01 vs. CT by unpaired Student’s *t-*test. Values are expressed as the mean ± SEM.

To determine the effect of FR on angiotensin receptors in a key RAS target tissue, we measured AT_1_R, AT_2_R and masR mRNA expression in mesenteric vessels. AT_1_R mRNA expression was 72% higher in the FR compared to CT group (Fig. 7A). In contrast, no differences between the FR and CT groups were observed in mRNA expression of the AT_2_R (Fig. 7B) or MasR (Fig. 6C).

### AT_1_R antagonist responses

**Figure 8:**
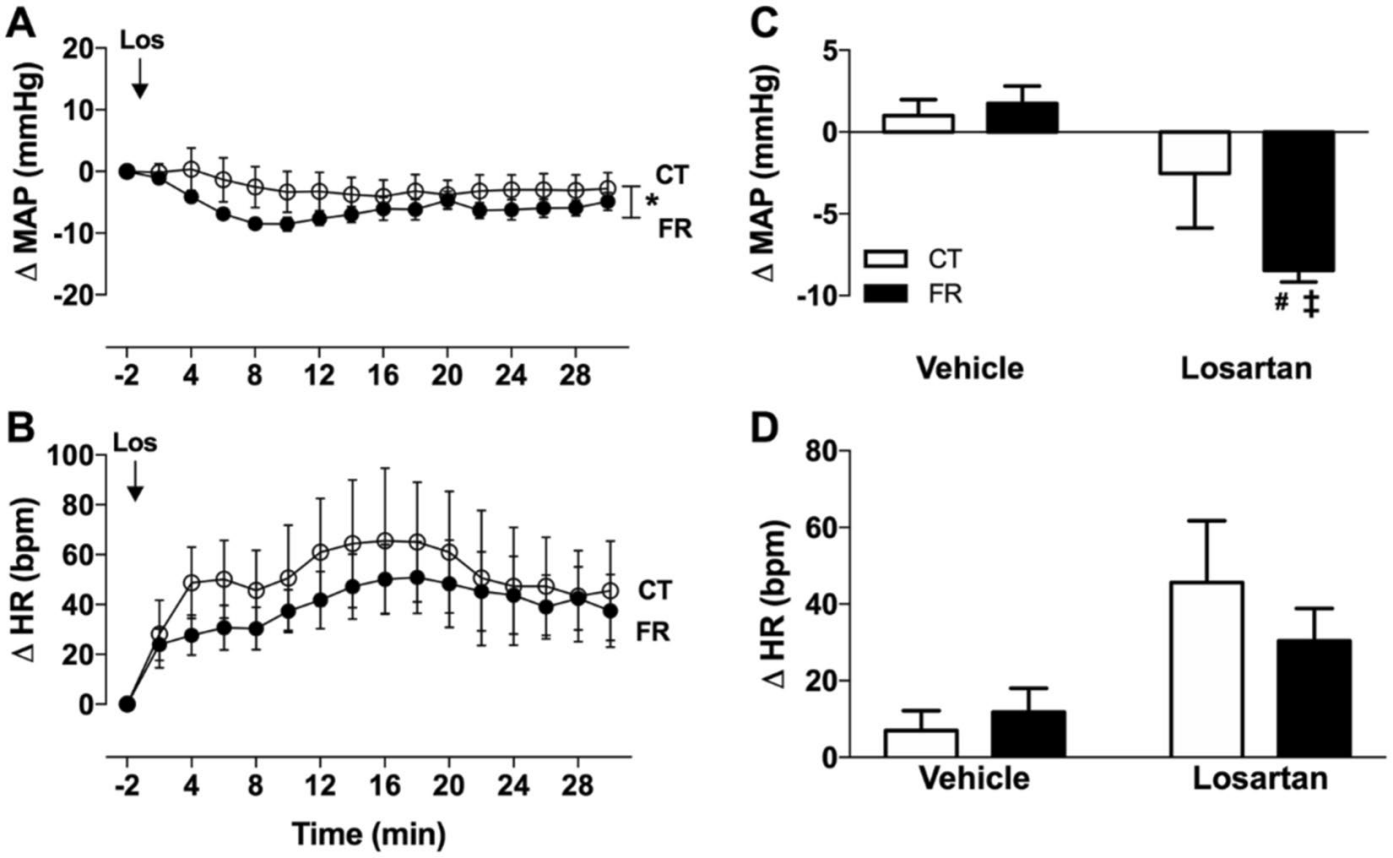
Effect of FR on MAP and HR responses to infusion of losartan. Shown are the changes (**A**,**B**) and maximum change from baseline (**C**,**D**) in MAP (**A**,**C**) and HR (**B**,**D**) after vehicle or losartan (Los) treatment in CT (open circle; white bar; n = 9) and FR (closed circle; black bar; n = 10) female rats. Basal MAP (mm Hg): CT, 122 ± 2 vs. *FR, 108 ± 1. Basal HR (bpm): CT, 374 ± 18 vs. FR, 365 ± 13. *P < 0.0001 vs. CT by two-way ANOVA and Bonferroni post-hoc test (time; diet); ^#^P < 0.05 vs. vehicle, same animal group by unpaired Student’s *t*-test and ^‡^p < 0.001 vs. CT, same treatment, by two-way ANOVA and Bonferroni post-hoc test (drug; diet); Values are expressed as the mean ± SEM.

To investigate the effect of FR on AT_1_R activity, we measured depressor responses to the AT_1_R antagonist, losartan. We found that the magnitude of the BP lowering effect of losartan was greater in the FR compared to CT group (Fig. 8A,C). FR had no effect on the HR responses to losartan (Fig. 8B,D).

### α1-adrenergic receptor agonist responses

**Figure 9:**
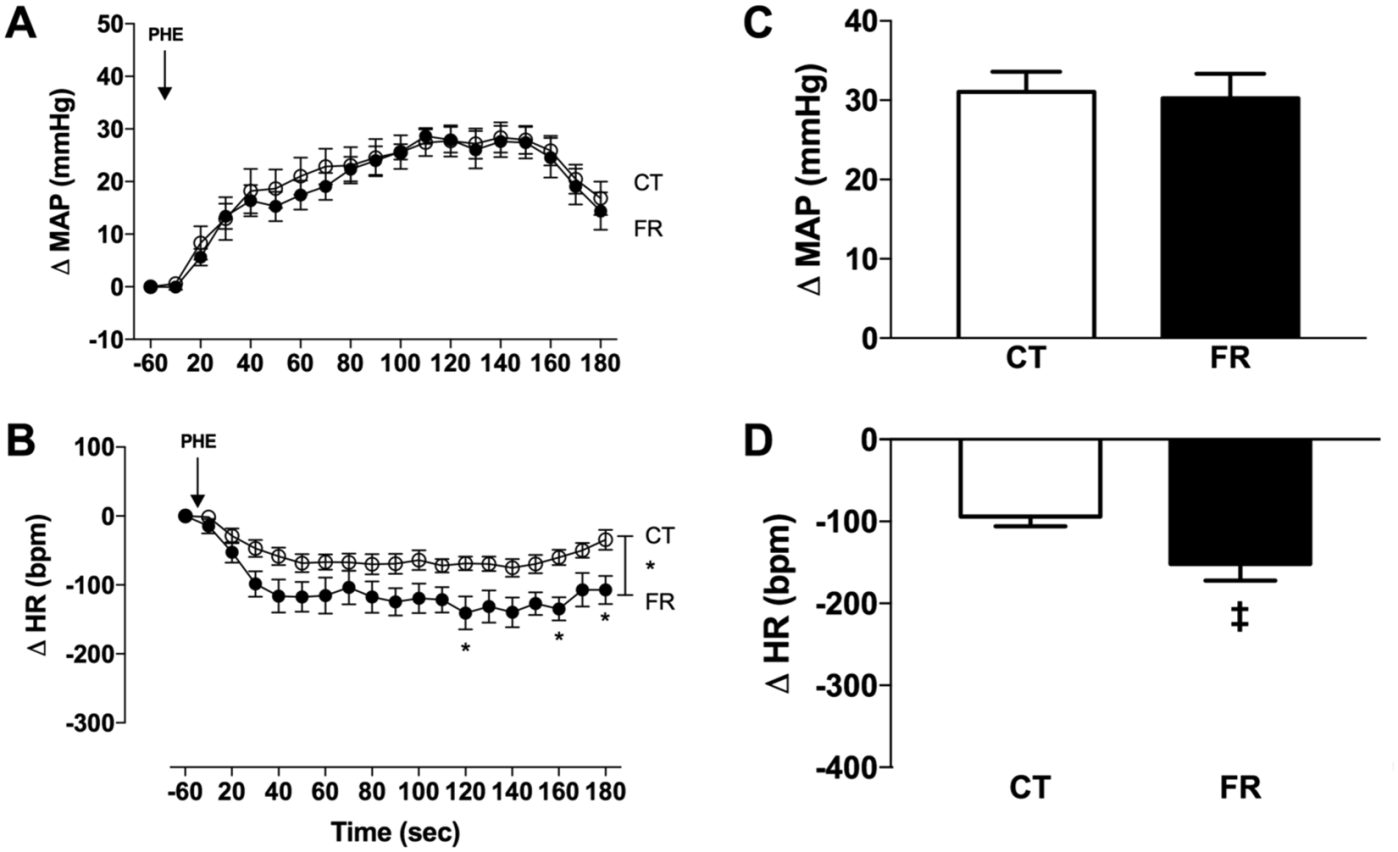
Effect of FR on MAP and HR responses to infusion of phenylephrine (PHE), an α-1 adrenergic receptor agonist. Shown are the changes (**A**,**B**) or maximum change from baseline (**C**,**D**) in MAP (**A**,**C**) and HR (**B**,**D**) after L-phenylephrine (299 nM/mL, infusion velocity: 2.2 mL/h) in CT (open circle) or FR (closed circle) rats (n = 9/group). Basal MAP (mm Hg): CT, 116 ± 3 vs. *FR, 106 ± 3. Basal HR (bpm): CT, 372 ± 15 vs. FR, 396 ± 14. *P < 0.001 vs. CT by two-way ANOVA and Bonferroni post-hoc test (time; diet); ^‡^P < 0.05 vs. CT, by unpaired Student’s *t-*test; Values are expressed as the mean ± SEM.

To determine if FR selectively altered pressor responses mediated by the RAS, we studied the effect of FR on pressor responses to the α1-adrenergic receptor agonist L-phenylephrine. We found that FR had no effects on the pressor response to L-phenylephrine (Fig. 9A,C); however, L-phenylephrine caused was a greater reduction in HR in the FR compared to CT group (P < 0.0001) (Fig. 9B,D).

## Discussion

A major finding of this study is that severe FR increased plasma Ang-[1–8] under conditions in which the animals remained hypotensive. Plasma Ang-[1–8] was increased primarily due to an increase in the conversion of AGT to Ang-[1–8] as a result of increased ACE activity and not renin. FR had no effect on plasma Ang-[1–10] or plasma renin activity even though AGT levels were higher in the FR compared to CT animals. While renin is well known to regulate plasma Ang-[1–8] through conversion of AGT to Ang-[1–10] and subsequent activation of ACE, previous studies in rat liver showed plasma Ang-[1–8] could also be regulated independently of renin directly through ACE^19^. Furthermore, studies have shown that AGT can also be degraded to Ang-[1–8] in renin-independent ways^20–23^. These findings indicate severe FR activates the RAS in a renin-independent manner.

A reduction in plasma volume is a well-known stimulus for Ang-[1–8] production^24^. When plasma volume is reduced by hemorrhage, Ang-[1–8] rises in the plasma causing increased vasoconstriction and water and sodium retention^25^. This activation of the RAS is a survival response, designed to counteract the drop in BP. Severe FR is likely to trigger this same mechanism since plasma volume is markedly reduced on the FR diet; however, even though the RAS is activated, the animal is unable to attain normal blood pressure due to the large drop in blood volume.

After infusion of Ang-[1–10], plasma AGT was reduced in the CT group. These findings support previous studies showing plasma AGT is reciprocally regulated by changes in plasma Ang-[1–8]^26,27^. In contrast, plasma AGT was up-regulated in the FR group after Ang-[1–10] infusion. Thus, the normal negative feedback response to the increase in plasma levels of Ang-[1–8] is either suspended or masked as a result of the need to compensate for the decrease in plasma volume.

Another major finding was that FR caused a coordinate up-regulation in both ACE and ACE2 activity. Many studies have shown ACE and ACE2 are regulated in a ying-yang manner. For example, ACE mRNA & protein expression was increased while ACE2 mRNA & protein expression was reduced in the paraventricular nucleus of the hypothalamus of the spontaneously hypertensive rat when compared to Wistar Kyoto normotensive controls^28^. However, other studies have found ACE and ACE2 can act coordinately. The mRNA & protein expression of both ACE and ACE2 were reduced by diabetic nephropathy in kidney tubules^29^. Although FR caused a coordinate up-regulation of activity of both the enzyme that produces Ang-[1–8] (i.e., ACE) and the enzyme that degrades Ang-[1–8] (i.e., ACE2), ACE is the predominant RAS catabolic pathway activated by the FR diet since plasma Ang-[1–7] was below the assay detection limit.

Few studies to date have investigated the effects of caloric or protein restriction on ACE and ACE2 activity; however, one study in ewes showed that restricting caloric intake by 30% in the periconceptional period increased the abundance of ACE protein and mRNA^30^. In contrast, individuals with AN were reported to have lower plasma ACE activity than age-matched controls^31^, however, the calorimetric method used to measure ACE in this human study has substrate specificity limitations that could impact the interpretation of the data. Therefore, it is not known whether the discrepancy between the human and animal data (rats and ewes) is due to species differences in the effects of FR on ACE or to methodological differences in assaying ACE activity.

Further evidence that the severe FR diet causes increased synthesis of plasma Ang-[1–8] is shown in the Ang-[1–10] infusion experiments. Ang-[1–10] caused a greater pressor response in the FR animals than CT group (Fig. 2). Ang-[1–10] can elicit a pressor response through its conversion to Ang-[1–8] by ACE. In fact, Ang-[1–10] infusion increased plasma Ang-[1–8] to a greater extent in the FR group (1.6-fold) than CT animals (1.3-fold) (Table 2). Not only was Ang-[1–8] up-regulated, Ang-[1–10] infusion led to higher plasma levels of Ang-[1–10] (5-fold) vs CT (3.5-fold) even though no differences in basal Ang-[1–10] were observed. The higher levels of plasma Ang-[1–10] in the FR group could be due to less catabolism or sequestration of plasma Ang-[1–10] than in the CT animals or to more Ang-[1–10] synthesis from endogenous AGT. In this regard, there was 20% more AGT under basal conditions in the FR group compared to CT animals (Table 2).

Alternatively, the decapeptide can cause a pressor response by conversion to another pressor peptide, Ang-[2–8], through the actions of aminopeptidase A and ACE independently of Ang-[1–8] formation^32^. We found that infusion of Ang-[1–10] in FR animals increased production of Ang-[2–8] by 1.4-fold, whereas no such increase in Ang-[2–8] was observed in the CT animals. These findings indicate that FR increased the activity of Ang-[1–8] metabolic pathways through ACE and possibly aminopeptidase A.

Ang-[1–8] mediates its physiological actions by binding to its receptors in target tissues including the vasculature. In the present study, we found that the FR diet up-regulated mRNA expression of the AT_1_R. Our findings are supported by a study showing that a protein restricted diet increased AT_1_R protein in rat aorta vessels^15^. This up-regulation of AT_1_R expression could be a compensatory response to the BP reduction induced by FR by serving to increase vasoconstriction in resistance vessels. This idea is supported by the AT_1_R antagonist infusion studies since we found losartan lowered BP to a greater extent in the FR animals compared to the CT group indicating that the activity of AT_1_Rs is greater in the FR rats. These results support a previous report showing that losartan caused a greater reduction in MAP in rats maintained on a low protein diet compared to controls^15^.

Given that the FR diet had no effect on AT_2_R or masR mRNA expression, it is unlikely that these receptors play a major role in the hypotension and bradycardia observed in this model. Only a few reports have investigated the effect of food or nutrient deficiency on AT_2_R expression and no studies to date have reported effects on the masR. One study in pregnant ewes fed a 50% nutrient-restricted diet during gestation found that the offspring were hypertensive and expressed high AT_2_R mRNA expression in the renal medulla^33^, however, the effect of nutrient restriction on AT_2_R expression in the mother was not investigated. Thus, it is not known whether the lack of effect of a FR diet on AT_2_R mRNA expression in mesenteric vessels is specific to rodents, target tissue differences (mesenteric vessels vs. renal medulla), the nature of the diet or other experimental differences.

The paradoxical effect of hypotension in the presence of increased plasma Ang-[1–8] and mesenteric AT_1_R expression is likely due to the inability of Ang-[1–8]-mediated vasoconstrictor mechanisms to overcome the reduction in MAP induced by low plasma volume. This idea is supported by the Ang-[1–8] infusion studies, which found Ang-[1–8] caused smaller increases in MAP in FR compared to CT animals (Fig. 3). The fact that basal plasma Ang II and mesenteric AT_1_R expression are both higher in FR rats than CTs raises the possibility that the AT_1_R is dysfunction and unable to cause sufficient vasoconstriction to raise BP.

Similar observations were seen in a study of patients with AN compared to age-matched controls; patients with AN had to be infused with a higher concentration of Ang-[1–8] to achieve a 20 mmHg increase in BP^17^. Furthermore, in a mouse model of hypotension induced by severe intravascular volume depletion and decreased renal Na^+^ reabsorption, aortic vessels exhibited decreased contractile reactivity^34^. This study suggested the cause of the reduced vasoconstriction was the reduced vascular hypertrophy.

An alternative interpretation of our findings is that the attenuated pressor response to Ang-[1–8] in the FR group is due to a ceiling effect on the ability of the vessel to vasoconstrict; however, this possibility would be AT_1_R specific since the vasoconstrictor, L-phenylephrine, increased MAP to a similar extent in the FR and CT animals. The normal BP response to Ang-[1–8] infusion is four-fold higher than to an equivalent dose of Ang-[1–10]^35,36^, which is similar to our findings in the control animals. In contrast, we found no differences in the pressor response in the FR animals between Ang-[1–10] and Ang-[1–8]. This attenuated pressor response to Ang-[1–8] may be due to impaired AT_1_R signaling even though AT_1_R mRNA expression in the FR animals was increased 2-fold in the mesenteric vessels. We were not able to measure AT_1_R protein expression because of the lack of AT_1_R antibody specificity nor could we detect AT_1_Rs by radioligand binding in mesenteric vessels. Thus, the caveat remains that the observed increased in AT_1_R mRNA expression may not reflect increased AT_1_R protein.

Another explanation for the lower Ang-[1–8] responsiveness in the FR group could be receptor desensitization. In a mouse model of sepsis, the animals developed hypotension and at the same time, the sensitivity of the kidney vasculature to Ang-[1–8] was reduced as a result of AT_1_R desensitization^37^. Another contributing factor could be nitric oxide since FR reduces plasma nitric oxide^9^ and lowering nitric oxide is associated with endothelial dysfunction. Moreover, increases in plasma Ang-[1–8] and AT_1_R expression are known to lower nitric oxide^38,39^.

Albumin plays a key role in controlling plasma osmolality besides plasma volume. Low plasma protein or sodium concentration will result in hypoosmolality causing water inside vessels to move into the intercellular space, resulting in edema and low blood volume^40^. Changes in blood osmolality and blood volume are well known to stimulate the vasoconstrictor arm of the RAS^41^. We previously showed that this FR model causes hypoalbuminemia^9,42^. Taken together, these studies suggest hypoalbuminemia contributes to both the hypotension and activation of the vasoconstrictor arm of the RAS in individuals experiencing inadequate levels of dietary protein as a result of AN, famine or other reasons^18^.

It is well known that the RAS can regulate the kidney function and BP. Another possible reason for the low BP in FR is due to the inability of the kidney to respond to the high circulating Ang-[1–8] levels. In animals with low plasma volume and intact RAS, Ang-[1–8] stimulates ALDO production and renal sodium and water reabsorption^43^. However, the FR rats in this study did not respond to the elevated Ang-[1–8] as evidenced by their net sodium balance (Table 2) and ALDO levels (Table 1). This indicates that kidneys of FR animals have become refractory to the actions of Ang-[1–8], the sum total of which results in reduced plasma volume and BP. One likely mechanism is desensitization of the AT_1_R, as it has been previously shown that mice with hypotension undergo renal AT_1_R desensitization^37^. Another result that can corroborate the possible AT_1_R desensitization is the normal basal plasma levels of ALDO in FR. It is well documented that Ang-[1–8] can act in the adrenal AT_1_R and release ALDO, which did not happened in FR rats. Another explanation for the lack of effect on plasma ALDO concentrations is a possible ceiling effect of calcium influx, which is necessary for the Ang-[1–8] ALDO release signaling^44^. Future studies will be needed to understand the mechanism on desensitization to Ang-[1–8] in FR.

The heptapeptide Ang-[1–7] has been shown to have vasodilator effects^45^; however, infusion of the peptide had no effect on MAP in the CT rats and only a small pressor effect was observed in the FR rats. Moreover, plasma levels of Ang-[1–7] after vehicle or Ang-[1–10] infusion were not detectable using the highly sensitive RAS-Fingerprint technique. These results suggest Ang-[1–7] is not a major contributor to BP homeostasis in this FR animal model.

As in patients with AN, HR was slower in the FR compared to CT animals. Bradycardia could be due to high parasympathetic output to the heart. Increased parasympathetic activation could explain the higher HR response after L-phenylephrine infusion in FR compared to CT animals due a change in the baroreflex. Furthermore, the HR response to Ang-[1–10] infusion was attenuated in the FR group. It is well known that in response to an acute increase in BP, HR is decreased by baroreflex stimulation in an attempt to bring BP back to normal^46^. Therefore, the Ang-[1–10]-induced increase in BP likely stimulates the baroreflex resulting in a compensatory decrease in HR.

In conclusion, FR reduces plasma volume and BP. In order to maintain peripheral blood perfusion, the pressor arm of the RAS is activated increasing AGT and Ang-[1–8] levels, ACE activity and AT_1_R responsiveness. However, the RAS upregulation is inadequate to achieve full BP homeostasis. RAS activation is limited by attenuated Ang-[1–8] pressor responses, negative feedback to AGT and impaired ALDO responsiveness. Furthermore, once the period of severe FR ends, it is not clear if the RAS remains activated. If so, this up-regulation of the RAS may contribute to the long term adverse cardiovascular consequences observed in individuals who currently have or previously suffered from FR either voluntarily or not. Thus, it will be important to further investigate the mechanisms by which FR resets the RAS and the long term adverse consequences to the cardiovascular system.

## Methods

### Ethical Approval

All procedures conducted in Brazil were approved by the Ethics Committee for Animal Research (CEUA-UFOP; no. - 2016/02) of the Universidade Federal de Ouro Preto. The Georgetown University Animal Care and Use Committee approved all protocols conducted in the USA (no. 16:1234). All experimental protocols and animal maintenance was performed in accordance with the NIH Guide for the Care and Use of Laboratory Animals, European Convention for the Protection of Vertebrate Animals used for Experimental and other Scientific Purposes and according to the journal policies and regulations on animal experimentation. All efforts were made to follow the 3 Rs (Replacement, Reduction and Refinement) and to avoid any unnecessary distress to the animals.

### Animals

All experiments were conducted on female Fischer rats initially weighing 180–190 g at 3 months of age. Studies were conducted at both the Federal University of Ouro Preto (UFOP, Brazil) and Georgetown University (GU, USA). In Brazil, animals were bred in the University Center of Animal Sciences, UFOP. In the USA, animals were purchased from Envigo Corp. (Frederick, MD). All animals were housed in individual cages on a 12 hr light-dark cycle at room temperature (24 °C). The same person (AS) conducted the studies at UFOP and at GU. All of the experiments involving BP measurements were done at UFOP and all the samples processed for biochemical and molecular analysis were done at GU. For all studies conducted at GU, we confirmed that the FR diet had similar effects on BW, food and water intake, tissue weight, the estrous cycle, BP, and HR as we observed at UFOP. We chose to focus on a female model of severe food restriction because there are more women than men who experience periods of severe food restriction due to self-imposed diets and eating disorders^3^. Furthermore, while many studies have investigated the effects of malnutrition during pregnancy on the offspring^4,5^ few have focused on the adverse consequences of severe FR to the mother *prior* to pregnancy.

### Diet

After receiving the animals, they stayed in the animal facility for 2 weeks to recover from potential travel stress and to enable acclimatization to the environment. After 2 weeks, the animals were single housed and food intake and BW were determined daily at 5:00 pm for each animal for two weeks. After this period, the animals were randomly divided into two groups: control (CT) or food-restricted (FR). The average food consumption determined for individual animals during the previous 2 weeks was used to calculate the amount of food given to the FR group, which was 40% of the average of their normal consumption (determined two weeks prior to the beginning of the study). BW was measured daily before replenishing the food, as we previously described^9^. The CT group had free access to food for the duration of the study period. All animals received a standard rat diet (Rodent diet 20, #5053, LabDiet, Marlborough, MA) and had *ad libitum* access to water.

### Catheter Implantation

After two weeks on CT or FR diets, the animals were subjected to catheter implantation, as described previously^47^. In brief, rats were anaesthetized (2.5% isoflurane at 3 L/min O_2_) and polyethylene catheters were inserted into the femoral artery for cardiovascular measurements and into the femoral vein for drug infusions. The catheters were tunneled subcutaneously and exteriorized at the back of the neck. After surgery, analgesics (4 mg/kg Ketoflex, 0.1 mL/300 g, s.c) and antibiotics (0.2 mL/100 g, penicillin, streptomycin, dihydrostreptomycin, s.c.) were administered post-surgery and 24 hrs after surgery if any sign of pain was observed. While the catheter inserted into the femoral artery could reduce blood flow to the lower leg, the rats quickly shift flow to collateral vessels to compensate for the loss of blood flow from the femoral artery and they recover well with minimum distress using this widely used method^9,10^. Experimental procedures began 48 h after recovery from the anesthesia. Separate animals were used for drug infusion and molecular studies, except the group that received Ang-[1–10] infusions since blood was collected from these animals to measure Ang peptides.

### MAP & HR

All experiments recording MAP and HR were made in conscious freely moving rats. The rat arterial catheter was connected to a pressure transducer (MLT0699; ADI Instruments, Bella Vista, Australia) and a signal amplifier (ETH-400; CB Sciences Inc., Milford, MA, USA). The analog signal from the amplifier was digitized by a 12-bit analog-to-digital converter (PowerLab/400; ADI Instruments), and the pulsatile arterial pressure was recorded at 1000 Hz using Chart 7.0 for Windows software (ADI Instruments). MAP and HR were derived on-line from the pulsatile arterial pressure measurements using pulse-to-pulse analysis^15^. Data for HR and MAP values were recorded continuously. Baseline MAP and HR were determined during the two min-period that preceded drug injections and are expressed as the mean ± SEM. Responses to treatments are calculated from continual 2 min averages after drug infusions and are expressed as the change in MAP or HR from baseline.

### Experimental design

Before the experiments were performed, the animals were brought to the experimental room in which the temperature was maintained at 24–25 °C. The animals stayed in their home cages for 2 h before the protocol started. All the following experiments were performed during the light cycle. The experiment commenced only after HR and MAP were stabilized for at least 10 min. All the acute infusions were made using a 0.5 mL Hamilton syringe (Hamilton Robotics, Reno, NV). The first experiment evaluated the influence of FR on Ang-[1–10] and ACE BP responses. Both CT and FR rats received an intravenous (i.v.) bolus infusion of vehicle (0.1 mL/100 g of BW). After 10 min, they were infused with Ang-[1–10] (154 nmol/L − 0.1 mL/100 g of BW) followed by recording the MAP and HR for 30 min. Captopril was infused on the second day via bolus intravenous injection (9.2 mmol/L − 0.1 mL/100 g of BW) instead of vehicle, followed by Ang-[1–10]. The first drug infused was randomized. The second series of experiments evaluated the influence of FR on Ang-[1–8] and losartan on MAP and HR responses. On the first day, vehicle or Ang-[1–8] (191 nmol/L − 0.1 mL/100 g of BW) was infused. One day later, vehicle or losartan (24 mmol/L − 0.1 mL/100 g of BW) was infused and cardiovascular responses were recorded for 30 min after each infusion or until baseline levels were reached. The third group of experiments evaluated the effects of FR on MAP and HR responses to Ang-[1–7]. In those experiments, the rats received vehicle or Ang-[1–7] (191 nmol/L − 0.1 mL/100 g of BW) by i.v. infusion in bolus followed by recording the cardiovascular responses for 40 min. The last experiment tested the BP amplitude responses to a non-angiotensin system agonist, i.e., the α1-adrenergegic receptor agonist, phenylephrine (299 nM/mL, infusion velocity: 2.2 mL/h). MAP and HR responses were recorded until they returned to baseline.

### Plasma Sodium and Potassium Concentration and Balance

This experimental group was acclimated to the metabolic cage for 48 hours beginning on day 11. On day 13, 24 h collections began. Rats were given *ad libitum* access to water and the amount of powdered rat chow (Rodent diet 20, #5053, LabDiet, Marlborough, MA - sodium 0.3% and potassium 1.10%) was given according to the diet protocol (see above), either *ad libitum* for CT rats (n = 7) or 40% of the CT diet for FR rats (n = 7). Food intake and urine volume were measured gravimetrically. At the end of the experiment, the animals were anaesthetized with isoflurane (2.5% isoflurane at 3 L/min O_2_) and the femoral artery and vein were catheterized for plasma volume measurements (see below). Blood was collected by abdominal aorta puncture and samples were centrifuged at 13,000 × g for 10 min to separate the plasma, which was stored in sterilized tubes at −80 °C until analysis. Sodium and potassium concentrations were determined by flame photometry (Model #2655-10; Cole-Parmer, Vernon Hills, IL).

### Plasma Volume

Plasma volume was measured by the Evan’s blue dye technique as previously described^48^. In brief, after baseline blood collection, Evan’s blue dye (0.3 mg/ml) was infused into the venous catheter. Blood collections (0.2 mL), were taken from the arterial catheter at 5 and 10 min post-infusion. Plasma was collected following centrifugation of whole blood. Standards were made in 1% plasma at 0, 1, 5, 10, and 20 g/ml of Evan’s blue dye. The concentration of Evan’s blue dye was measured in a 100 μL plasma sample using a plate reader (FLUOstar Omega, BMG Labtech Inc., Cary, NC) at 610 nm at baseline before dye administration and at 5- and 10-min following dye administration. Background corrections were made by subtracting the observed concentration at baseline from the observed concentrations of the 5- and 10-min time points. Average plasma volumes were calculated by dividing the amount of administered Evan’s blue dye (75 g) by the background corrected concentrations.

### Plasma AGT

Parallel animal groups were used for all plasma and urine measurements except plasma volume and angiotensin peptide concentrations. Plasma concentrations of AGT were measured using an ELISA kit (Immuno-Biological Laboratories, Minneapolis, MS)^49^. In brief, 100 μl/well of standard rodent AGT (0.08–5.0 ng/mL) or plasma samples (1: 2,500 diluted in ELISA buffer) were added to the ELISA plate and incubated at 37 °C for 1 h. After the incubation, the plates were rinsed seven times with Washing Buffer provided by the kit. The plates were then incubated with 100 μl/well of horseradish peroxidase-labeled C-terminal antibody (1:30 diluted in antibody solution) at 37 °C for 30 min. After the incubation, the plates were rinsed nine times with Washing Buffer. Next, the plates were incubated with 100 μl/well of 3,3′,5,5′-tetramethylbenzidine solution under light-protected conditions at room temperature for 30 min. Finally, 100 μl of sulfuric acid (0.5 mol/l) was added to each well to stop the reaction. The absorbance values were measured at 450 nm.

### Plasma ALDO

Plasma ALDO was measured by ELISA (Enzo Life Sciences, NY). 100 μL of plasma diluted 1:5 was added to the plate immediately after the addition of antibody (50 μL) and the samples, the plate was incubated overnight at 4 °C. The next day, the plate was washed 3 times and 200 μL of substrate was added followed by a 1 h incubation at room temperature. The reaction was stopped by the addition of trisodium phosphate solution. The absorbance values were measured at 405 nm.

### RAS-Fingerprint®

After 14 days on the CT or FR diet, the rats were anesthetized with Inactin (80 mg/Kg) and infused (iv) with vehicle (0.2 mL) or Ang-[1–10] (154 nmol/L, 0.2 mL). After 10 min, whole blood was collected by cardiac puncture using a syringe filled with heparin and a proprietary protease inhibitor cocktail from Attoquant Diagnostics (Vienna, Austria). Plasma was obtained by centrifugation (2000 × *g*, 4 °C, 10 min) and stored at −80 °C until use. Circulating angiotensin peptide concentrations were determined by mass spectrometry using plasma samples collected in the presence of an inhibitor cocktail containing broad spectrum inhibitors against aspartic proteases (pepstatin A), cysteine proteases (p-hydroxymercuribenzoic acid), metalloproteases (ethylenediaminetetraacetic acid, 1,10-phenanthroline), serine proteases (AEBSF) and specific inhibitors for renin and aminopeptidases A and N at a final concentration of 5% v/v (Attoquant Diagnostics) and which completely blocked angiotensin metabolism. Stabilized samples were further spiked with stable isotope-labeled internal standards for each angiotensin metabolite including Ang-[1–10], Ang-[1–9], Ang-[1–8], Ang-[1–7], Ang-[1–5], Ang-[2–10], Ang-[2–8], Ang-[2–7], Ang-[3–8], and Ang-[3–7] at a concentration of 200 pg/ml. Following C18-based solid-phase-extraction, samples were subjected to LC-MS/MS analysis using a reversed-phase analytical column (Acquity UPLC® C18, Waters, Milford, MA) operating in line with a XEVO TQ-S triple quadrupole mass spectrometer (Waters) in multiple reaction monitoring mode. Internal standards were used to correct for peptide recovery of the sample preparation procedure for each angiotensin metabolite in each individual sample. Angiotensin peptide concentrations were calculated considering the corresponding response factors determined in appropriate calibration curves in original sample matrix, on condition that integrated signals exceeded a signal-to-noise ratio of 10.

### ACE Activity

Plasma ACE activity was measured as previously described^50^ with a few adaptations using the fluorogenic substrate Abz-Phe-Arg-Lys(Dnp)-Pro-OH. Reactions were conducted in 96 well microtiter plates containing 80 μL of Reaction Buffer (1 mol/L NaCl, 0.5 mmol/L ZnCl_2_, 75 mmol/L Tris, pH 7.5) in the presence of vehicle or the ACE inhibitor, captopril (20 µmol/L). Total enzyme activity was measured in the presence of vehicle (Reaction Buffer). Non-ACE activity was defined as peptidase activity measured in the presence of captopril. Specific ACE activity was defined as ACE activity minus non-ACE activity. Immediately after adding 10 μL of fluorogenic substrate, 10 µL of plasma, diluted 10× times, was added to each well to reach a final concentration of 60 μmol/L substrate. Product formation was determined at 37 °C by following the fluorescence as a function of time using a fluorescence plate reader (FLUOstar Omega) at an excitation wavelength of 320 nm and an emission wavelength of 410 nm. Initial velocities were determined from the rate of fluorescence increase over the 10–60 min time course corresponding to the linear range of the assay. Enzyme kinetics were analyzed using Prism 7.0 (GraphPad Software Inc, La Jolla, CA) (n = 8–9/group).

### ACE2 Activity

Plasma ACE2 activity was measured as we described previously^51^ using the fluorogenic substrate Mca-Ala-Pro-Lys(Dnp)-OH. Reactions were conducted in 96 well microtiter plates containing 85 μL of Reaction Buffer in the presence of vehicle or the ACE inhibitor, captopril (20 µmol/L) and the ACE2 inhibitor MLN-4760 (20 µmol/L). Total enzyme activity was measured in the presence of vehicle (Reaction Buffer). Non-ACE activity was defined as peptidase activity measured in the presence of captopril (20 µmol/L). Nonspecific peptidase activity was defined as the enzyme activity measured in the presence of captopril and MLN-4760. Specific ACE2 activity was defined as non-ACE activity minus nonspecific peptidase activity. Immediately after adding 10 μL of fluorogenic substrate, 5 μL of plasma was added to each well to reach a final concentration of 30 μmol/L substrate. Product formation was determined at 37 °C by following the fluorescence as a function of time using a fluorescence plate reader (FLUOstar Omega) at an excitation wavelength of 320 nm and an emission wavelength of 410 nm. Initial velocities were determined from the rate of fluorescence increase over the 10–100 min time course corresponding to the linear range of the assay. Enzyme kinetics were determined using Prism software as described above (n = 8/group).

### PRA

Plasma renin activity was measured using the 5-FAM/QXL^®^520 fluorescence resonance energy transfer (FRET) peptide^52^. Reactions were conducted in 96 well microtiter plates containing 40 μL of plasma and the product formation was determined at 37 °C by following the fluorescence as a function of time using a fluorescence plate reader (FLUOstar Omega) at an excitation wavelength of 490 nm and an emission wavelength of 520 nm. Data were recorded at 2 min intervals and initial velocities were determined from the rate of fluorescence increased over the 10–100 min time course, which corresponded to the linear range of the assay. The data were expressed as the rate of change in fluorescence, using the product of 5-carboxyfluorescein (5-FAM) as a marker for PRA (n = 10–8/group).

### qPCR

The mesenteric cascade, including the superior mesenteric artery was dissected from the intestinal wall using ice cold phosphate buffered saline (PBS) and this tissue was removed from the same rat used for plasma measurements. After the fat and veins were removed from the vessels using an Olympus dissecting microscope, the tissue was snap frozen in a dry ice-methanol bath and stored at −80 °C until use. The mesenteric vessels were homogenized using ceramic microbeads and total RNA was extracted using RNeasy according to the manufacturer’s instructions (Bio-Rad). Purified RNA (1 μg) was reverse transcribed with a high-capacity cDNA reverse transcription kit. Quantitation of specific rRNA was performed by real-time PCR using the ABI Prism 7700 Sequence Detection System (Applied Biosystems, Foster City, CA). The PCR reaction mixture consisted of RNase-free water, SYBR green supermix and 300 nmol/L specific primers as previously described^53^: angiotensin type 1a receptor (AT_1a_R) **-** F: 5′-CTC AAG CCT GTC TAC GAA AAT GAG-3′; R: 5′-TAG ATC CTG AGG CAG GGT GAA T-3′; angiotensin type 2 receptor (AT_2_R) **-** F: 5′-ACC TTT TGA ACA TGG TGC TTT G-3′; R: 5′-GTT TCT CTG GGT CTG TTT GCT C-3′; MAS oncogene (Mas) **-** F: 5′-CACTGGCCCTCCTGATGAA-3′; R 5′-GGATGCCAGAATTGAACACAGA-3′; and, beta-actin **-** F: 5′-CCCATCTATGAGGGTTACGC-3′, R – 5′-TTTAATGTCACGCACGATTTC-3′. The tissue levels of these cDNAs were calculated based on the standard curves.

### Reagents

Ang-[1–10], Ang-[1–8], Ang-[1–7], captopril (Cap), losartan, inactin, L-phenylephrine and Evans blue dye were purchased from Sigma (St. Louis, MO). Isoflurane was purchased from Cristália Ltda (Itapira, SP, Brazil). Ketoprofen (Ketoflex) was purchased from Mundo Animal (Pindamonhangaba, SP, Brazil). Penicillin, streptomycin, and dihydrostreptomycin were purchased from Fort Dodge Animal Health (Hialeah, FL, USA). Abz-Phe-Arg-Lys(Dnp)-Pro-OH was purchased from GenScript (Piscataway, NJ, USA). Mca-Ala-Pro-Lys(Dnp)-OH was purchased from Enzo Life Science (Farmingdale, NY, USA). MLN-4760 was purchased from EMD Milllipore (Billerica, MA, USA). Ceramic microbeads were purchased from MP Biomedicals (Solon, OH, USA). RNeasy was purchased from Qiagen (Germantown, MD, USA). RNase-free water, SYBR green supermix, and high-capacity cDNA reverse transcription kits (#1708890) were purchased from Bio-Rad (Hercules, CA, USA). Renin activity 520 Rat Renin Fluorimetric assay kit was purchased from SensoLyte (AnaSpec, San Jose-CA). Angiotensinogen Elisa kit was purchased from IBL (Hamburg, Germany). Aldosterone Elisa kit was purchased from Enzo Life Science (Farmingdale, NY, USA).

### Statistical Analysis

Prism 7.0 (GraphPad Software, La Jolla, CA, USA) was used to analyze all data and to construct the graphs. The data are expressed as mean ± standard error of the mean (SEM). The data for basal parameters characterization, enzyme activity, mRNA expression and peptide concentrations were analyzed first for normality using the Shapiro-Wilk normality test and when following the normality, were analyzed using the Student’s unpaired *t*-test to assess differences between groups. All the results expressed as a curve after drug stimulation over time were compared by two-way (time and diet as factors) analysis of variance (ANOVA) followed by Bonferroni post-test using all the time-points showed in the graph. To test treatment effects within the same group, the Student’s *t*-test was used when the data followed a normal distribution. The significance threshold level was set at 0.05.
